# Patient characteristics and initiation of mineralocorticoid receptor antagonists in patients with chronic kidney disease in routine clinical practice in the US: a retrospective cohort study

**DOI:** 10.1186/s12882-019-1348-4

**Published:** 2019-05-16

**Authors:** Michael Blankenburg, Anne-Kathrin Fett, Seline Eisenring, Gabriele Haas, Alain Gay

**Affiliations:** 1 Bayer AG, Market Access, Pharmaceuticals, Berlin, Germany; 2 IQVIA Commercial GmbH & Co. OHG, Unterschweinstiege 2-14, 60549 Frankfurt/Main, Germany; 3 IQVIA, Theaterstrasse 4, 4051 Basel, Switzerland; 4 Bayer AG, Medical Affairs, Pharmaceuticals, 13342 Berlin, Germany

**Keywords:** Chronic kidney disease, Type 2 diabetes, Heart failure, Mineralocorticoid receptor antagonist, Real-world treatment patterns

## Abstract

**Background:**

Steroidal mineralocorticoid receptor antagonists (MRAs) are recommended for the treatment of heart failure (HF) and resistant hypertension, both common comorbidities in patients with diabetes and chronic kidney disease (CKD). This study explored the clinical characteristics of, and steroidal MRA use in, patients with CKD with and without type 2 diabetes mellitus (T2D) and/or HF.

**Methods:**

This retrospective cohort study used PharMetrics Plus US claims database data (October 2009–September 2014) to identify two patient populations aged ≥18 years with a first diagnosis of CKD or a first prescription for steroidal MRAs. Demographic characteristics, comorbidities, clinical events, medication use and healthcare costs were reported by population and stratified by diagnosis: CKD, CKD + T2D (DKD), CKD + HF and DKD + HF. The CKD population cohorts were further stratified by steroidal MRA treatment duration (no MRAs, < 6 and ≥ 6 months’ treatment).

**Results:**

The CKD and MRA populations comprised 229,004 patients and 5899 patients, respectively. Median age and the proportion of men were similar in the CKD and MRA populations across disease cohorts. Disease burden increased across the cohorts as comorbidity and clinical event incidences increased. Hypertension was reported in 70–92% of patients, irrespective of disease cohort or population. In the CKD population, MRA use was low but increased with disease burden: CKD, 1.2%; DKD, 1.8%; CKD + HF, 6.5%; and DKD + HF, 6.6%. Moreover, MRA users presented with higher rates of comorbidities and medication use, and higher healthcare costs than MRA non-users. Longer MRA treatment duration was associated with reduced polypharmacy, lower event rates and lower healthcare costs. In the MRA population, patients almost exclusively received spironolactone (≥ 96%; median dose across all groups 25 mg; one-year persistence, ≤ 43%); up to 16% of patients had end-stage renal disease at baseline despite steroidal MRAs being contraindicated.

**Conclusions:**

Steroidal MRA use was low across all cohorts, but increased with disease severity, driven particularly by HF. Steroidal MRAs were used in patients with advanced CKD, despite being contraindicated. The persistent morbidity and clinical event rates in CKD and DKD patients highlight the disease burden and the need for treatments that effectively target both cardio-vascular and kidney-related events.

**Electronic supplementary material:**

The online version of this article (10.1186/s12882-019-1348-4) contains supplementary material, which is available to authorized users.

## Background

Chronic kidney disease (CKD) is associated with a gradual, progressive loss of kidney function. It is classified into five stages of severity, culminating in end-stage renal disease (ESRD) [1, 2]. The prevalence of CKD was recently estimated to be 11–13% globally, with moderate (stage 3) CKD being the most common [3]. CKD is often co-incident with several chronic conditions including obesity, diabetes, hypertension and heart failure (HF) [4–6]. The rising prevalence of obesity and diabetes worldwide, particularly in low- to middle-income countries, has further increased the burden of CKD to society [4, 6]. The presence of type 2 diabetes mellitus (T2D) is the leading cause of ESRD, and the presence of CKD in patients with T2D has been shown to increase cardiovascular morbidity and mortality dramatically [6–8].

Overactivation of the mineralocorticoid receptor present in both cardiac and renal cells in response to elevated aldosterone levels, high salt load, increased plasma glucose or increased reactive oxygen species generation plays an important role in cardiovascular disease and CKD [9, 10]. Available research suggests that the steroidal mineralocorticoid receptor antagonists (MRAs), spironolactone and eplerenone, reduce blood pressure, especially in resistant hypertension, reduce cardiovascular mortality and hospitalizations in HF and improve albuminuria excretion in CKD [9–16]. However, the potential benefit of steroidal MRAs is limited by numerous adverse reactions, including hyperkalemia and worsening renal function [11, 17, 18].

Previous research has not investigated the association between steroidal MRAs and therapeutic outcomes in patients with CKD and various comorbid conditions. This study was conducted to explore real-world steroidal MRA use and clinical characteristics of the respective patient populations and to identify predictors of MRA use in these populations.

## Methods

### Study design

This retrospective, non-interventional cohort study examined patient characteristics and steroidal MRA use over a five-year observation period (October 2009–September 2014). The data source for this analysis was the PharMetrics Plus United States (US) claims database (PMTX+). PMTX+ comprises the adjudicated claims of more than 150 million patients. Diagnoses and procedures were coded to US claims standards (International Classification of Diseases, Ninth Revision, Clinical Modification [ICD-9-CM] at the time of the study), Current Procedural Terminology and Healthcare Common Procedure Coding System. Drug treatment was captured using records of filled prescriptions (National Drug Code and Generic Product Identification) and recorded utilization in medical settings. Healthcare costs included amounts allowed by health plans. Patient records were anonymized in compliance with the Health Insurance Portability and Accountability Act 1996.

#### Patient population

**Fig. 1:**
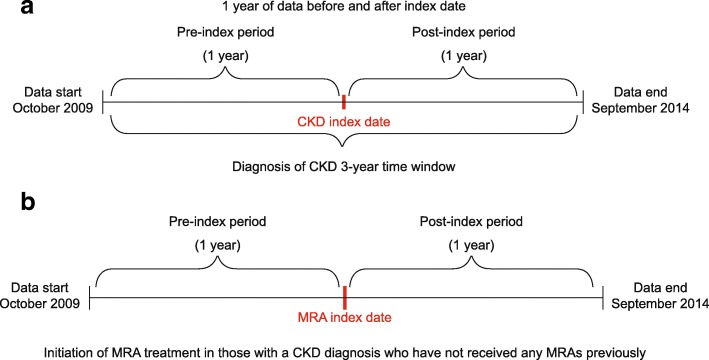
Study design showing the respective (**a**) CKD and (**b**) MRA populations *CKD* chronic kidney disease, *MRA* mineralocorticoid receptor antagonist

Two populations were investigated in this study and were not mutually exclusive; some patients were included in both populations if they met the respective criteria. The CKD population comprised patients who were aged at least 18 years and who had received a diagnosis of CKD after the start of the observation period in a time window that allowed for at least one year of data coverage before the diagnosis date (inclusion date) and for at least one year of data coverage after this date (Fig. 1). This included patients diagnosed for the first time and those who may have received a repeat diagnosis. Eligible patients may have received previous treatment with steroidal MRAs before this time window. This population was used to investigate characteristics of patients with and without steroidal MRA treatment and predictors of steroidal MRA initiation.

The MRA population comprised patients who were at least 18 years of age, with a diagnosis of CKD, and who received a first prescription for a steroidal MRA (spironolactone or eplerenone) after the start of the observation period in a time window that allowed for at least one year of data observation before the first prescription date (inclusion date) and for at least one year of data observation after this date (Fig. 1). In this population, the diagnosis of CKD could have occurred at any time before or during the overall observation period. This population was used for analyses of MRA dose and treatment persistence.

Using ICD-9-CM diagnosis codes (Additional file 1: Table S1), participants in each population were stratified into one of the following disease cohorts: CKD only (CKD), CKD with T2D (this combination was considered a proxy for diabetic kidney disease [DKD] in this study), CKD with HF (CKD + HF) or CKD with T2D and HF (DKD + HF).

### Study objectives

The primary objective of the study was to describe the clinical characteristics of patients with CKD with and without HF and/or T2D, and the real-world treatment patterns, including steroidal MRA initiation, in these patient cohorts. The secondary objective was to evaluate clinical predictors of steroidal MRA initiation.

### Variables

Baseline variables (present at inclusion data or up to 12 months before) assessed in both populations included demographics, CKD stage (ICD-9-CM), comorbidities (based on ICD-9-CM codes), concomitant medication use and healthcare costs. It should be noted that ICD-9-CM codes differentiate between stage 5 CKD and ESRD based on a requirement for chronic dialysis. Follow-up variables included concomitant medication, clinical events use and healthcare costs.

The following variables were only assessed in the CKD population: previous steroidal MRA use, proportion of patients initiating steroidal MRAs and time to initiation of steroidal MRA treatment. The following variables were only assessed in the MRA population: dosing of steroidal MRAs and persistence on steroidal MRA therapy.

### Statistical analyses

Descriptive analyses were performed for all baseline variables. For categorical measures, numbers of cases and percentages are reported. For continuous variables, the mean value with 95% confidence interval, standard deviation and median are reported.

Statistical comparisons across groups are reported at baseline only. Χ^2^ tests were used for categorical variables and Wilcoxon rank-sum tests were used for continuous variables.

Clinical events, based on ICD-9-CM codes (Additional file 1: Table S2), and medication use during follow-up, based on GPI codes, are reported by diagnostic group and MRA treatment condition (no MRAs, < 6 and ≥ 6 months’ treatment) to capture characteristics by treatment duration.

The analysis of predictors of steroidal MRA treatment and clinical outcomes was conducted by means of logistic regression, overall and within each cohort. Given the exploratory nature of the study, all potential predictors of interest were initially included in the model, with final reported predictors selected by running step-wise logistic regression. Age and sex were always included. All statistical analyses were performed using SAS 9.1.3 (SAS Institute, Cary, NC, USA).

## Results

### Patient demographics and baseline characteristics in the CKD population

**Fig. 2:**
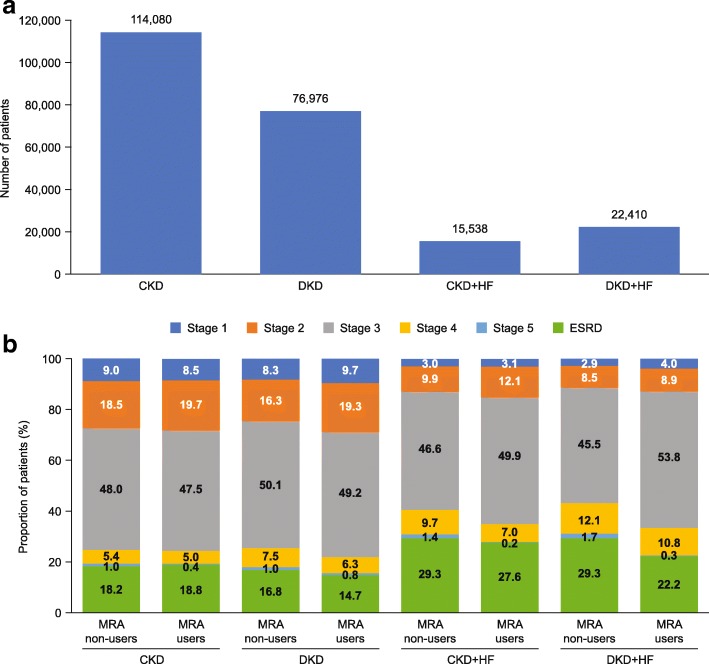
(**a**) Patient distribution and (**b**) CKD stage distribution (where reported) across the study cohorts in the CKD population *CKD* chronic kidney disease, *DKD* diabetic kidney disease, ESRD *end-stage renal disease, HF* heart failure, *MRA* mineralocorticoid receptor antagonist

In total, 229,004 patients had a diagnosis of CKD during the study period and were eligible for inclusion. Of these, 114,080 patients had CKD only, 76,976 patients had DKD, 15,538 patients had CKD with HF and 22,410 patients had DKD with HF (Fig. 2a).

Table 1 shows baseline characteristics for each disease cohort stratified by MRA initiation. Median age increased, from 59 years in the CKD cohort of MRA non-users to 64 years in the DKD + HF MRA non-users. In each cohort, the median age was lower in MRA users than in MRA non-users.

**Table 1:** Baseline demographics and characteristics of the CKD population by cohort and post-initiation MRA treatment duration

	CKD cohort						DKD						CKD + HF						DKD + HF					
	No MRA		Spiro < 6 months		Spiro ≥ 6 months		No MRA		Spiro < 6 months		Spiro ≥ 6 months		No MRA		Spiro < 6 months		Spiro ≥ 6 months		No MRA		Spiro < 6 months		Spiro ≥ 6 months	
	*n* = 112,730		*n* = 869		*n* = 481		*n* = 75,616		*N* = 891		*N* = 469		*N* = 14,653		*N* = 512		*N* = 373		*N* = 21,144		*N* = 808		*N* = 458	
Age, years																								
Mean (SD)	58.4		56.8		57.2		61.5		59.4		58.8		66.7		62.0		62.6		65.5		63.5		63.4	
Median	59.0		57.0		57.0		61.0		60.0		59.0		67.0		61.0		62.0		64.0		62.0		62.0	
Range	18 to 83		19 to 83		21 to 83		18 to 83		21 to 83		23 to 83		18 to 83		22 to 83		22 to 83		18 to 83		27 to 83		32 to 83	
Male, *n* (%)	62,284	(55.3)	403	(46.4)	224	(46.6)	44,790	(59.2)	508	(57.0)	286	(61.0)	8527	(58.2)	336	(65.6)	253	(67.8)	12,789	(60.5)	515	(63.7)	312	(68.1)
Physician specialty, *n* (%)																								
Primary care Physician	21,585	(19.1)	122	(14.0)	72	(15.0)	13,203	(17.5)	129	(14.5)	40	(8.5)	1850	(12.6)	38	(7.4)	37	(9.9)	2228	(10.5)	72	(8.9)	30	(6.6)
Internal medicine	24,512	(21.7)	146	(16.8)	80	(16.6)	15,970	(21.1)	157	(17.6)	94	(20.0)	2622	(17.9)	74	(14.5)	37	(9.9)	3533	(16.7)	126	(15.6)	75	(16.4)
Cardiology	4160	(3.7)	26	(3.0)	20	(4.2)	2774	(3.7)	24	(2.7)	12	(2.6)	1103	(7.5)	41	(8.0)	19	(5.1)	1182	(5.6)	57	(7.1)	33	(7.2)
Nephrology	26,862	(23.8)	251	(28.9)	154	(32.0)	18,608	(24.6)	298	(33.4)	169	(36.0)	2436	(16.6)	71	(13.9)	61	(16.4)	4646	(22.0)	150	(18.6)	85	(18.6)
Hospital	11,941	(10.6)	120	(13.8)	67	(13.9)	9110	(12.0)	122	(13.7)	59	(12.6)	2744	(18.7)	137	(26.8)	122	(32.7)	4148	(19.6)	204	(25.2)	128	(27.9)
Other	21,346	(18.9)	175	(20.1)	74	(15.4)	14,304	(18.9)	144	(16.2)	79	(16.8)	3563	(24.3)	135	(26.4)	87	(23.3)	4882	(23.1)	172	(21.3)	95	(20.7)
Unknown	2324	(2.1)	29	(3.3)	14	(2.9)	1647	(2.2)	17	(1.9)	16	(3.4)	335	(2.3)	16	(3.1)	10	(2.7)	525	(2.5)	27	(3.3)	12	(2.6)
Previous use of steroidal MRAs																								
Spironolactone	2949	(2.6)	0	(0.0)	0	(0.0)	2900	(3.8)	0	(0.0)	0	(0.0)	1774	(12.1)	0	(0.0)	0	(0.0)	3087	(14.6)	0	(0.0)	0	(0.0)
Eplerenone	174	(0.2)	0	(0.0)	0	(0.0)	159	(0.2)	0	(0.0)	0	(0.0)	97	(0.7)	0	(0.0)	0	(0.0)	144	(0.7)	0	(0.0)	0	(0.0)
Medications, *n* (%)																								
Angiotensin II receptor blockers	21,797	(19.3)	256	(29.5)	155	(32.2)	21,943	(29.0)	339	(38.0)	198	(42.2)	3036	(20.7)	125	(24.4)	89	(23.9)	5874	(27.8)	251	(31.1)	145	(31.7)
ACE inhibitors	34,066	(30.2)	295	(33.9)	169	(35.1)	34,146	(45.2)	438	(49.2)	248	(52.9)	5445	(37.2)	223	(43.6)	151	(40.5)	9565	(45.2)	400	(49.5)	237	(51.7)
Renin inhibitors	737	(0.7)	14	(1.6)	13	(2.7)	1052	(1.4)	25	(2.8)	17	(3.6)	116	(0.8)	7	(1.4)	2	(0.5)	343	(1.6)	19	(2.4)	8	(1.7)
β-blockers	33,078	(29.3)	351	(40.4)	198	(41.2)	30,472	(40.3)	468	(52.5)	285	(60.8)	8297	(56.6)	316	(61.7)	225	(60.3)	13,715	(64.9)	531	(65.7)	321	(70.1)
Calcium- channel blockers	24,880	(22.1)	308	(35.4)	177	(36.8)	22,904	(30.3)	377	(42.3)	215	(45.8)	4238	(28.9)	137	(26.8)	108	(29.0)	7493	(35.4)	279	(34.5)	175	(38.2)
Vasodilators	11,115	(9.9)	164	(18.9)	117	(24.3)	12,443	(16.5)	233	(26.2)	123	(26.2)	3512	(24.0)	142	(27.7)	105	(28.2)	7228	(34.2)	259	(32.1)	162	(35.4)
Diuretics	25,848	(22.9)	363	(41.8)	228	(47.4)	25,907	(34.3)	477	(53.5)	259	(55.2)	7291	(49.8)	313	(61.1)	227	(60.9)	12,900	(61.0)	580	(71.8)	320	(69.9)
Comorbidities, *n* (%)																								
Hypertension	78,431	(69.6)	672	(77.3)	381	(79.2)	65,617	(86.8)	801	(89.9)	425	(90.6)	12,209	(83.3)	420	(82.0)	275	(73.7)	19,502	(92.2)	727	(90.0)	417	(91.0)
CVD	14,518	(12.9)	133	(15.3)	72	(15.0)	15,907	(21.0)	224	(25.1)	96	(20.5)	7166	(48.9)	266	(52.0)	176	(47.2)	11,645	(55.1)	438	(54.2)	250	(54.6)
IHD	14,234	(12.6)	115	(13.2)	70	(14.6)	17,319	(22.9)	221	(24.8)	89	(19.0)	6932	(47.3)	233	(45.5)	163	(43.7)	12,440	(58.8)	462	(57.2)	267	(58.3)
LVH	3163	(2.8)	35	(4.0)	23	(4.8)	3150	(4.2)	75	(8.4)	31	(6.6)	2819	(19.2)	143	(27.9)	102	(27.3)	4632	(21.9)	198	(24.5)	110	(24.0)
Anemia	23,359	(20.7)	241	(27.7)	110	(22.9)	21,008	(27.8)	240	(26.9)	126	(26.9)	5616	(38.3)	157	(30.7)	111	(29.8)	9710	(45.9)	289	(35.8)	168	(36.7)
Hyperkalemia	3257	(2.9)	32	(3.7)	12	(2.5)	4496	(5.9)	37	(4.2)	18	(3.8)	1079	(7.4)	18	(3.5)	12	(3.2)	2671	(12.6)	61	(7.5)	28	(6.1)
Healthcare costs, $																								
Mean	18,160		23,803		21,729		24,659		28,455		24,404		53,565		53,905		46,850		66,119		54,849		55,348	
95% CI	17,878 to 18,442		20,351 to 27,256		17,699 to 25,759		24,263 to 25,054		24,362 to 32,548		20,706 to 28,103		51,882 to 55,248		44,942 to 62,868		37,678 to 56,022		64,579 to 67,659		48,251 to 61,448		46,433 to 64,263	
SD	48,268		51,857		44,981		55,491		62,246		40,763		103,912		103,231		90,084		114,247		95,549		97,083	
Median	5720		8120		7558		10,059		11,758		12,535		19,661		20,854		16,934		29,288		25,929		24,633	
Time to first treatment, days^a^																								
Mean	–		167.6		52.7		–		177.8		53.0		–		139.1		55.2		–		146.2		54.6	
95% CI	–		159.7 to 175.6		47.9 to 57.6		–		170.2 to 185.4		48.0 to 57.9		–		128.9 to 149.2		49.9 to 60.5		–		138.2 to 154.3		49.6 to 59.6	
SD	–		119.1		54.5		–		115.3		54.7		–		117.4		52.4		–		11,116.8		52.5	
Median	–		184.0		32.0		–		196.0		33.0		–		118.5		37.0		–		123.0		35.0	

*CI* confidence interval, *CKD* chronic kidney disease, *CVD* cardiovascular disease, *DKD* diabetic kidney disease, *ESRD* end-stage renal disease, *GP* general practitioner, *HF* heart failure, *HMO* health maintenance organization, *IHD* ischemic heart disease, *LVH* left ventricular hypertrophy, *PPO* preferred provider organization, *MRA* mineralocorticoid receptor antagonist, *SD* standard deviation, *Spiro* spironolactone

^a^No pre-inclusion MRA use

Data on CKD stage was not available for approximately one-third of patients across all cohorts. For patients for whom information on CKD stage was available (N = 153,407/229,004), stage 3 CKD was the most common stage identified at baseline, irrespective of disease cohort or MRA use (45.5–50.1% for MRA non-users and 47.5–53.8% for MRA users). Patients with HF were more likely to be at a higher stage of CKD than those without HF (Fig. 2b). The proportion of patients with ESRD was higher in the cohorts with HF than in those without HF (Fig. 2b); it was also higher in the patients who received steroidal MRA treatment for up to six months than who received steroidal MRA treatment for at least six months (Table 1).

Previous use (more than 12 months before inclusion date) of spironolactone across the disease cohorts was low but increased along the disease cohorts from CKD to DKD + HF: CKD, 2.6%; DKD, 3.8%; CKD + HF, 12.1%; and DKD + HF, 14.6%. Thus, steroidal MRA use appeared to associate with disease burden. Previous use of eplerenone was very low across all cohorts (574/224,143 of MRA non-users). Owing to the low number of patients receiving eplerenone, only data from patients receiving spironolactone are reported as MRA users.

While concomitant medication use increased across the disease cohorts in line with the presence of T2D and HF, there were some differences in the pattern of use. For example, use of angiotensin II receptor blockers (ARBs) and angiotensin-converting enzyme (ACE) inhibitors appeared to be driven by the presence of T2D but not of HF. In contrast, the use of β-blockers, vasodilators, diuretics, renin inhibitors, and calcium-channel blockers appeared to be driven by the presence of both T2D and HF (Table 1). The proportion of patients who were using sodium polystyrene sulfonate, a potassium binder prescribed for the treatment of hyperkalemia, was low irrespective of cohort, ranging from 0.7–1.3% for non-users of MRAs and from 0.0–7.1% of MRA users; the highest use was observed in patients with DKD without HF who had used MRAs for less than 6 months.

For the comorbidities of hypertension, CVD, IHD, LVH and anemia, there was a trend for the presence of increasing number of comorbidities along the disease cohorts CKD, DKD, CKD + HF, and DKD + HF.

### Initiation of steroidal MRAs in the CKD population

The number of patients in the CKD population who were initiated on spironolactone during the study period was low for all cohorts but was higher for patients with HF than for those without HF: CKD 1350/114,080 (1.2%); DKD 1360/76,976 (1.8%); CKD + HF 885/15,538 (5.7%); and DKD + HF 1266/22,410 (5.6%). Across the cohorts, the mean time to steroidal MRA initiation following CKD diagnosis ranged from 52.7 to 55.2 days in those receiving treatment for at least six months and from 139.1 to 177.8 days for those receiving treatment for less than six months (Table 1).

Steroidal MRA therapy was most commonly initiated by specialists, with nephrologists being the most common prescribers in those with CKD or DKD and without HF (Table 1).

### Predictors of steroidal MRA use in the CKD population

The logistic regression analysis of predictors of steroidal MRA use is summarized in Table 2. The following predictors were consistently associated with steroidal MRA initiation across all cohorts: previous medication with ARBs or ACE inhibitors and presence of comorbid edema. Prescription by a specialist clinician at inclusion rather than a primary care physician was also associated with steroidal MRA initiation; however, the type of specialty that showed a significant association varied depending on the cohort.

**Table 2:** Logistic regression of predictors for MRA initiation by cohort in the CKD population

Independent variables	CKD				DKD				CKD + HF				DKD + HF			
	Odds ratio	Confidence interval		*P* value	Odds ratio	Confidence interval		P value	Odds ratio	Confidence interval		P value	Odds ratio	Confidence interval		P value
		Lower limit	Upper limit			Lower limit	Upper limit			Lower limit	Upper limit			Lower limit	Upper limit	
Age, years (vs 18–34 years)																
35–44	1.93	1.43	2.61	< 0.0001	1.15	0.72	1.82	0.5666	0.82	0.50	1.34	0.4255	1.10	0.47	2.53	0.83
45–54	1.48	1.11	1.96	0.0075	1.01	0.66	1.55	0.9672	0.98	0.64	1.51	0.9423	1.31	0.60	2.85	0.50
55–64	1.31	0.99	1.73	0.0593	0.90	0.59	1.37	0.6131	0.70	0.46	1.06	0.0897	1.14	0.53	2.48	0.73
65+	0.95	0.71	1.27	0.7419	0.63	0.41	0.98	0.0381	0.40	0.27	0.61	< 0.0001	0.80	0.37	1.74	0.58
Sex (vs male)																
Female	1.43	1.28	1.59	< 0.0001	1.04	0.93	1.16	0.4554	0.77	0.66	0.89	0.0004	0.83	0.73	0.93	0.002
Prescribing physician specialty at inclusion (vs general/family practice)																
Internal Medicine	1.04	0.86	1.26	0.695	1.25	1.03	1.52	0.0266	1.03	0.77	1.39	0.8268	1.30	1.02	1.66	0.03
Cardiology	1.28	0.93	1.75	0.126	1.11	0.78	1.59	0.5677	1.36	0.97	1.92	0.0789	1.79	1.34	2.40	< 0.0001
Nephrology	1.45	1.22	1.73	< 0.0001	1.76	1.47	2.10	< 0.0001	1.27	0.95	1.70	0.1068	1.11	0.88	1.41	0.38
Hospital	1.69	1.38	2.07	< 0.0001	1.49	1.21	1.85	0.0002	2.09	1.61	2.73	< 0.0001	1.68	1.34	2.11	< 0.0001
Other/unknown	1.34	1.12	1.601	0.0017	1.24	1.02	1.51	0.0288	1.50	1.15	1.95	0.0027	1.30	1.03	1.63	0.02
Previous medication use (yes vs no)																
ARBs	1.75	1.55	1.97	< 0.0001	1.62	1.44	1.82	< 0.0001	1.40	1.19	1.65	< 0.0001	1.34	1.18	1.52	< 0.0001
ACE inhibitors	1.24	1.10	1.39	0.0003	1.37	1.22	1.54	< 0.0001	1.33	1.16	1.54	< 0.0001	1.34	1.19	1.51	< 0.0001
Renin inhibitors	2.11	1.44	3.10	0.0001	1.66	1.21	2.27	0.0016	1.28	0.65	2.52	0.476	1.25	0.83	1.890	0.28
Vasodilators	2.20	1.92	2.53	< 0.0001	1.64	1.45	1.86	< 0.0001	1.25	1.06	1.46	0.0066	0.97	0.85	1.09	0.59
*Comorbidities*																
LVH	1.26	0.97	1.65	0.0884	1.71	1.39	2.09	< 0.0001	1.71	1.46	2.000	< 0.0001	1.28	1.11	1.46	0.0005
Anemia	1.07	0.94	1.21	0.2916	0.84	0.74	0.95	0.0063	0.72	0.62	0.84	< 0.0001	0.70	0.62	0.79	< 0.0001
Edema	2.14	1.85	2.47	< 0.0001	2.09	1.83	2.38	< 0.0001	1.48	1.26	1.73	< 0.0001	1.47	1.30	1.66	< 0.0001
Proteinuria	1.18	1.00	1.41	0.0571	1.31	1.13	1.51	0.0004	0.82	0.59	1.15	0.2475	0.98	0.80	1.20	0.85
Hyperkalemia	0.96	0.71	1.30	0.8046	0.55	0.42	0.73	< 0.0001	0.49	0.34	0.70	0.0001	0.57	0.45	0.71	< 0.0001
Sensitivity testing: c-statistic	0.67				0.66				0.68				0.63			

*ACE* acetylcholinesterase, *ARB* angiotensin II receptor blocker, *CKD* chronic kidney disease, *DKD* diabetic kidney disease, *HF* heart failure, *LVH* left ventricular hypertrophy, *MRA* mineralocorticoid receptor antagonist

The presence of hyperkalemia or left ventricular hypertrophy (both determined by ICD-9-CM codes; Additional file 1: Table S2) was significantly associated with lower odds of steroidal MRA initiation for all cohorts except the CKD cohort. Being a woman was predictive of steroidal MRA use in all cohorts except the DKD cohort. The associations between age and steroidal MRA use differed across the disease cohorts. Being middle aged (35–44 years or 45–54 years) rather than younger (18–34 years) was significantly associated with higher odds of steroidal MRA use in the CKD cohort; an age of 65+ years versus 18–34 years was significantly associated with higher odds of steroidal MRA use in the DKD and CKD + HF cohorts. Age was unrelated to the odds of steroidal MRA use in the DKD + HF cohort. The concordance statistics for the full model across the four cohorts are reported in Table 2.

### Concomitant medication use, incidence of clinical events and healthcare costs during follow-up

#### Concomitant medication use during follow-up

The use of concomitant medications during the follow-up period by disease cohort, stratified by steroidal MRA use is summarized in Table 3. ACE inhibitors were used by 30.2–45.2% of MRA non-users and by 33.9–52.5% of MRA users across the cohorts, depending on treatment duration. A similar pattern was seen with ARB prescriptions (Table 3). Use of diuretics was higher in MRA users compared with MRA non-users across all cohorts, and was highest in patients who had received steroidal MRA treatment for less than six months; a similar pattern was seen for vasodilators (Table 3).

#### Clinical events during follow up

The incidence of myocardial infarction increased across the disease cohorts, driven primarily by the presence of HF and to a lesser extent by the presence of T2D (3.1–19.4%). In addition, the incidence of myocardial infarction was higher in MRA users than in MRA non-users; this increase occurred irrespective of steroidal MRA treatment duration in the presence of HF (6.8–27.0%).

The incidence of stroke was also primarily driven by the presence of HF and less so by T2D (8.7–25.1%). This pattern was also observed in all MRA users, with increased incidences when treatment was for short duration (Table 3).

**Table 3:** Concomitant medication and clinical events during follow-up by cohort and MRA treatment duration in the CKD population

Characteristics	CKD						DKD						CKD + HF						DKD + HF					
	No MRA		Spironolactone				No MRA < 6 months		Spironolactone				No MRA		Spironolactone				No MRA		Spironolactone			
			< 6 months		≥ 6 months				< 6 months		< 6 months				< 6 months		≥ 6 months				< 6 months		≥ 6 months	
	(*n* = 112,730)		(*n* = 869)		(*n* = 481)		(*n* = 75,616)		(*n* = 891)		(*n* = 469)		(*n* = 14,653)		(*n* = 512)		(*n* = 373)		(*n* = 21,144)		(n = 808)		(*n* = 458)	
Concomitant medications, *n* (%)																								
Angiotensin II receptor blockers	23,232	(20.6)	293	(33.7)	163	(33.9)	22,675	(30.0)	365	(41.0)	194	(41.4)	2915	(19.9)	134	(26.2)	88	(23.6)	5402	(25.5)	248	(30.7)	134	(29.3)
ACE inhibitors	33,843	(30.0)	322	(37.1)	145	(30.1)	33,084	(43.8)	435	(48.8)	221	(47.1)	5149	(35.1)	265	(51.8)	191	(51.2)	8486	(40.1)	447	(55.3)	232	(50.7)
Renin inhibitors	671	(0.6)	18	(2.1)	9	(1.9)	889	(1.2)	23	(2.6)	13	(2.8)	99	(0.7)	8	(1.6)	0	(0.0)	245	(1.2)	18	(2.2)	9	(2.0
β-blockers	36,496	(32.4)	515	(59.3)	261	(54.3)	33,657	(44.5)	611	(68.6)	328	(69.9)	9176	(62.6)	434	(84.8)	323	(86.6)	14,537	(68.8)	706	(87.4)	406	(88.6)
Calcium-channel blockers	29,305	(26.0)	417	(48.0)	217	(45.1)	26,359	(34.9)	477	(53.5)	253	(53.9)	4625	(31.6)	172	(33.6)	122	(32.7)	7820	(37.0)	351	(43.4)	169	(36.9)
Vasodilators	12,863	(11.4)	263	(30.3)	126	(26.2)	14,483	(19.2)	349	(39.2)	168	(35.8)	3960	(27.0)	224	(43.8)	145	(38.9)	7827	(37.0)	405	(50.1)	223	(48.7)
Diuretics	26,812	(23.8)	526	(60.5)	255	(53.0)	27,196	(36.0)	627	(70.4)	315	(67.2)	7647	(52.2)	432	(84.4)	302	(81.0)	13,023	(61.6)	714	(88.4)	385	(84.1)
Clinical events, *n* (%)																								
Reproductive system and breast disorders^a^	10,517	(9.3)	78	(9.0)	59	(12.3)	7028	(9.3)	117	(13.1)	59	(12.6)	1032	(7.0)	39	(7.6)	29	(7.8)	1532	(7.2)	72	(8.9)	39	(8.5)
Hyperkalemia	5187	(4.6)	94	(10.8)	53	(11.0)	6890	(9.1)	149	(16.7)	67	(14.3)	1593	(10.9)	78	(15.2)	53	(14.2)	3647	(17.2)	190	(23.5)	75	(16.4)
Stroke	9821	(8.7)	120	(13.8)	42	(8.7)	9532	(12.6)	179	(20.1)	63	(13.4)	3249	(22.2)	125	(24.4)	78	(20.9)	5307	(25.1)	219	(27.1)	99	(21.6)
Myocardial infarction	3440	(3.1)	59	(6.8)	26	(5.4)	3975	(5.3)	105	(11.8)	35	(7.5)	2185	(14.9)	118	(23.0)	90	(24.1)	4098	(19.4)	218	(27.0)	124	(27.1)
Heart failure	3066	(2.7)	99	(11.4)	30	(6.2)	4418	(5.8)	203	(22.8)	39	(8.3)	11,274	(76.9)	470	(91.8)	351	(94.1)	16,511	(78.1)	765	(94.7)	425	(92.8)
Thrombosis	3594	(3.2)	51	(5.9)	26	(5.4)	2708	(3.6)	58	(6.5)	28	(6.0)	1246	(8.5)	72	(14.1)	32	(8.6)	1834	(8.7)	89	(11.0)	39	(8.5)

*ACE* acetylcholinesterase, *ARB* angiotensin II receptor blocker, *CKD* chronic kidney disease, *DKD* diabetic kidney disease, *HF* heart failure, *LVH* left ventricular hypertrophy, *MRA* mineralocorticoid receptor antagonist

^a^Physical feminization, breast tenderness, gynecomastia, testicular atrophy, reversible infertility, loss of libido and erectile dysfunction, menstrual irregularities

The incidence of reported hyperkalemia increased across the disease cohorts, driven equally strongly by the presence of HF and of T2D (4.6–17.2%). It was higher in MRA users, especially in patients with a short duration of steroidal MRA use (10.8–23.5%) (Table 3).

#### Healthcare costs

Median healthcare costs in MRA non-users ranged from $7473 in patients with CKD to $38,885 in those with DKD + HF. Median costs were higher for patients receiving MRAs for at least six months (range, $11,960–$51,525) than for MRA non-users. The highest median costs were seen for patients receiving steroidal MRAs for less than six months (ranging from $18,015 for patients with CKD to $66,910 for those with DKD + HF) (Additional file 1: Table S3).

### Patient demographics and baseline characteristics in the MRA population

**Fig. 3:**
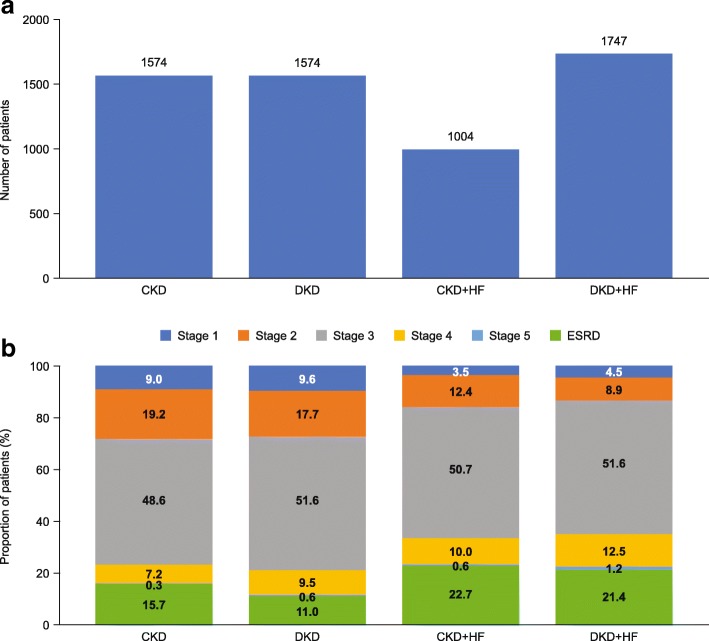
(**a**) Patient distribution and (**b**) CKD stage distribution (where reported) across the study cohorts in the MRA population *CKD chronic kidney disease,* DKD *diabetic kidney disease,* ESRD *end-stage renal disease,* HF *heart failure,* MRA *mineralocorticoid receptor antagonist*

In total, 5899 patients were included in the MRA population. The respective breakdown across the disease cohorts is shown in Fig. 3a. When compared to the distribution of patients in the CKD population (Fig. 2a), there was a more even distribution of patients across the four disease cohorts in the MRA population, reflecting increased steroidal MRA use driven by HF. Table 4 shows the baseline characteristics for each disease cohort in the MRA population. Median age increased with presence of T2D and/or HF, ranging from 57.0 years to 63.0 years. The proportion of males ranged from 47.5 to 66.8% across the disease cohorts. The proportion of patients with ESRD at baseline ranged from 8.8 to 15.7%, despite steroidal MRAs being contraindicated in this population (Fig. 3b and Table 4).

**Table 4:** Baseline characteristics of the MRA population by disease cohort

Variable	MRA cohort							
	CKD		DKD		CKD + HF		DKD + HF	
	(*n* = 1574)		(*n* = 1574)		(*n* = 1004)		(*n* = 1747)	
Age, years								
Mean (SD)	56.1 (13.0)		59.0 (10.6)		62.7 (13.5)		63.5 (10.7)	
Median	57.0		60.0		61.0		63.0	
Range	19 to 83		22 to 83		22 to 83		28 to 83	
Men, *n* (%)	747	(47.5)	925	(58.8)	671	(66.8)	1113	(63.7)
Physician specialty, *n* (%)								
Primary care physician	207	(13.2)	166	(10.5)	106	(10.6)	173	(9.9)
Internal medicine	258	(16.4)	280	(17.8)	163	(16.2)	310	(17.7)
Cardiology	48	(3.0)	37	(2.4)	94	(9.4)	128	(7.3)
Nephrology	602	(38.2)	666	(42.3)	210	(20.9)	430	(24.6)
Hospital	176	(11.2)	152	(9.7)	190	(18.9)	295	(16.9)
Other	241	(15.3)	235	(14.9)	211	(21.0)	370	(21.2)
Unknown	42	(2.7)	38	(2.4)	30	(3.0)	41	(2.3)
Medications, *n* (%)								
Angiotensin II receptor blockers	539	(34.2)	704	(44.7)	258	(25.7)	573	(32.8)
ACE inhibitors	609	(38.7)	817	(51.9)	460	(45.8)	924	(52.9)
Renin inhibitors	46	(2.9)	55	(3.5)	13	(1.3)	50	(2.9)
Β-blockers	735	(46.7)	993	(63.1)	710	(70.7)	1366	(78.2)
Calcium-channel blockers	722	(45.9)	817	(51.9)	321	(32.0)	766	(43.8)
Vasodilators	395	(25.1)	529	(33.6)	351	(35.0)	789	(45.2)
Diuretics	778	(49.4)	953	(60.5)	694	(69.1)	1390	(79.6)
Comorbidities, *n* (%)								
Hypertension	1391	(88.4)	1516	(96.3)	949	(94.5)	1696	(97.1)
CVD	275	(17.5)	412	(26.2)	641	(63.8)	1145	(65.5)
IHD	245	(15.6)	388	(24.7)	595	(59.3)	1212	(69.4)
LVH	106	(6.7)	156	(9.9)	390	(38.8)	668	(38.2)
Anemia	518	(32.9)	576	(36.6)	436	(43.4)	941	(53.9)
Hyperkalemia	95	(6.0)	109	(6.9)	75	(7.5)	216	(12.4)
Healthcare costs, $								
Mean	31,380		32,761		77,274		84,733	
95% CI	28,275 to 34,485		29,995 to 35,527		69,997 to 84,551		79,324 to 90,141	
SD	62,801		55,954		117,501		115,256	
Median	11,944		16,185		38,447		48,117	

*ACE* acetylcholinesterase, *ARB* angiotensin II receptor blocker, *CKD* chronic kidney disease, *DKD* diabetic kidney disease, *HF* heart failure, *LVH* left ventricular hypertrophy, *m* months, *MRA* mineralocorticoid receptor antagonist

Similar patterns in concomitant medication use at baseline were observed in the MRA population (Table 4) when compared with the CKD population (Table 1).

The most common comorbidity at inclusion in the MRA population was hypertension (88.4–97.1%) (Table 4). The incidence of comorbidities (e.g. CVD, IHD, LVH, anemia, edema) in the MRA population was predominantly higher in the presence of HF (Table 4).

### MRA dose and persistence in the MRA population

**Fig. 4:**
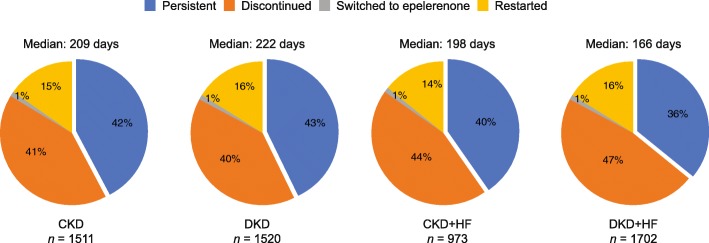
Spironolactone persistence during the one-year follow up by disease cohort in the MRA population *CKD chronic kidney disease, DKD diabetic kidney disease, HF* heart failure, *MRA* mineralocorticoid receptor antagonist

Patients in the MRA population were almost exclusively treated with spironolactone (≥ 96%); the median dose of spironolactone prescribed was 25 mg (Additional file 1: Table S4). Of those few patients (2.6–4.0%) who were prescribed eplerenone the median dose was 50 mg in the non-HF cohorts and 25 mg in the cohorts with HF. One-year persistence with spironolactone ranged from 36 to 43%. Only 1% of patients across all cohorts switched from spironolactone to eplerenone (Fig. 4).

## Discussion

To our knowledge, this retrospective, exploratory study is the first to describe the respective patient characteristics and use of steroidal MRAs in routine clinical practice for four distinct cohorts of patients, with CKD, DKD, CKD with HF, or DKD with HF.

### Patient characteristics and use of MRAs in the CKD and MRA population studied

At least 70% of patients in the CKD population and at least 88% of patients in the MRA population reported hypertension. Steroidal MRA use was at least threefold higher in patients with HF than in those without HF, suggesting that most prescribing of steroidal MRAs was in line with current guidelines recommending their use in patients with hypertension or heart failure (New York Heart Association [NYHA] Class 3–4 and left ventricular ejection fraction ≤35%) [15, 16, 19, 20]. However, given the relatively low proportions of patients in the CKD population that were subsequently prescribed steroidal MRAs (less than 6%), our results suggest that steroidal MRA use is rare in clinical practice and are in line with other reports showing that steroidal MRA use is low even in guideline-eligible patients [21, 22]. Reassuringly, given the guideline recommendations for steroidal MRA use, fewer than 5% of patients in the MRA population lacked a recorded code for either hypertension or HF; this may reflect missing data rather than the absence of the condition.

Of the available steroidal MRAs, patients predominantly received spironolactone, which could be either due to lower costs or its greater effectiveness compared with eplerenone [23–26]. However, fewer than half of the patients were still receiving steroidal MRA treatment one year post-initiation; it is possible that this is be linked to the incidence of adverse events such as hyperkalemia, but the exact reasons for discontinuation were not available in the database.

As might be predicted, steroidal MRA therapy was most commonly initiated by specialists, particularly nephrologists, cardiologists and other hospital specialists, irrespective of the population studied, suggesting its use often occurs later in the development of disease. This most likely reflects the recommendation of steroidal MRAs as fourth-line therapy for hypertension in the guidelines [27, 28]. Indeed, patients who received steroidal MRAs were more likely to be multimorbid and more advanced in chronic kidney disease, as well as having higher medication loads and healthcare costs than the overall CKD population. Interestingly, steroidal MRAs were also prescribed to a proportion of patients with stage 4 and 5 CKD, or ESRD, for which they are contra-indicated. [19, 20]

### Predictors of steroidal MRA initiation in the CKD population

Being seen by a specialist, previous treatment with ARBs or ACE inhibitors, previous steroidal MRA use, and multimorbid conditions were all significant predictors of steroidal MRA initiation in the CKD population. This reflects an increased prescription of steroidal MRAs in those patients with higher disease severity, particularly those with hypertension or HF. Moreover, there were differences between disease cohorts in the type of specialist that was predictive of steroidal MRA prescription. As might be expected, nephrologists were predictive for steroidal MRA initiation in CKD and DKD cohorts, but not for those with HF; internal medicine specialists were only predictive for steroidal MRA intiation in patients with DKD, while cardiologists were associated with an increased likelihood to prescribe steroidal MRAs in the most complex patient cohort (DKD + HF).

### Clinical events, concomitant medication use and healthcare costs in the CKD population

In general, steroidal MRA prescription was associated with the presence of more comorbid conditions and with higher rates of clinical events. Steroidal MRA use for less than six months was associated with a higher prevalence of all clinical events, including hyperkalemia, stroke, and myocardial infarction, when compared with steroidal MRA use for more than six months, suggesting that these events manifest early and may contribute to the decision to discontinue treatment. While the current study does not indicate any association between steroidal MRA use and the incidence of any of the clinical events reported, previous research assessing hyperkalemia risk observed higher risk estimates for short-term usage of steroidal MRAs compared with long-term usage [29].

As would be expected, given that steroidal MRA use is associated with more complex disease status, healthcare costs were higher for MRA users than for MRA non-users, and higher for MRA users with shorter rather than longer treatment durations. These observations reflect the inherent characteristics of the treatment groups, with patients with a more complex morbidity status requiring more care in terms of medication, hospitalization and outpatient visits. Systematic, longitudinal research will be necessary to investigate to what extent steroidal MRAs can influence healthcare utilization and costs.

### Study strengths and limitations

The main strength of this longitudinal study is the inclusion of real-world clinical practice data covering a large number of patients who were eligible for inclusion across all four disease cohorts of interest. Moreover, records in the PMTX+ database are representative of the national, commercially insured, real-world population in terms of age and sex. In addition, the use of a large claims database removes the potential for selection or physician bias. However, the results from this study do need to be viewed in light of several limitations of using a claims database. For example, the geographic coverage of the PTMX+ database does not fully reflect the US census population; older patients are underrepresented in the data set and the use of a US data source may not allow for generalization to other countries. Moreover, because patients’ complete medical history is not available, it is possible that the date of first CKD diagnosis, or of first MRA use, could include repeated as well as new diagnoses. Also, the disease cohort “DKD” was built by combining CKD and T2D codes, which is an approximation that falls short of a true diagnosis of DKD. Similarly, because the full treatment history could not be assessed, certain values may be missing and the reason for a given prescription cannot always be directly ascertained. Moreover, due to the nature of the database, information on patient mortality was not collected in this study; therefore, potential immortality bias could not be considered in the analysis. Finally, the exclusion of previous users of MRAs and use of just one year of follow-up to assess MRA treatment persistence precludes conclusions about potential differences between long-term and newly initiated users of MRAs.

## Conclusions

The study shows that CKD patients with T2D and/or HF and higher rates of clinical events (e.g. MI or stroke) are more likely to receive steroidal MRAs. Patients with CKD who received steroidal MRAs tended to have an increased disease severity, defined by comorbidities and elevated clinical event rates, and to have complex poly-pharmaceutical treatment regimens. Steroidal MRAs therefore appear to be indicators of advanced disease states; however, the limited use and treatment persistence observed in this study, suggest that alternative treatments with improved patient tolerance would be desirable for the management of CKD and DKD.

## Additional file

Additional file 1: **Table S1.** ICD-9-CM pre-inclusion diagnosis codes **Table S2.** ICD-9-CM codes for comorbidities **Table S3.** Total healthcare costs (US$) during follow-up by cohort and MRA treatment condition in the CKD **Table S4.** Treatment and dosage at inclusion date in the MRA cohort. (DOCX 50 kb)

## Abbreviations

ACE: Angiotensin-converting enzyme
ARB: Angiotensin receptor II blocker
CI: Confidence interval
CKD: Chronic kidney disease
CVD: Cardiovascular disease
DKD: Diabetic kidney disease
ESRD: End-stage renal disease
GP: General practitioner
HF: Heart failure
HMO: Health maintenance organization
ICD-9-CM: International Classification of Diseases, Ninth Revision, Clinical Modification
IHD: Ischemic heart disease
LVH: Left ventricular hypertrophy
MRA: mineralocorticoid receptor antagonist
PMTX+: PharMetrics Plus US claims database
PPO: Preferred Provider Organization
SD: Standard deviation
Spiro: Spironolactone
T2D: Type 2 diabetes

## References

[1] Meguid El Nahas A, Bello AK (2005). Chronic kidney disease: the global challenge. Lancet..

[2] United States Renal Data System 2016 Annual Data Report. Available at: https://www.usrds.org/2016/view/Default.aspx. Accessed 3 May 2019.

[3] Hill NR, Fatoba ST, Oke JL, Hirst JA, O’Callaghan CA, Lasserson DS (2016). Global prevalence of chronic kidney disease – a systematic review and meta-analysis. PLoS One.

[4] Thomas MC, Cooper ME, Zimmet P (2015). Changing epidemiology of type 2 diabetes mellitus and associated chronic kidney disease. Nat Rev Nephrol.

[5] Kovesdy CP, Furth SL, Zoccali C (2017). Obesity and kidney disease: hidden consequences of the epidemic. Br J Med Biol Res.

[6] Gansevoort RT, Correa-Rotter R, Hemmelgarn BR, Jafar TH, Heerspink HJL, Mann JF, Matsushita K, et al. Chronic kidney disease and cardiovascular risk: epidemiology, mechanisms, and prevention. Lancet. 2013;382:339–52. 10.1016/S0140-6736(13)60595-4 23727170

[7] Matsushita K, van der Velde M, Astor BC, Woodward M, Levey AS, de Jong PE (2010). Association of estimated glomerular filtration rate and albuminuria with all-cause and cardiovascular mortality: a collaborative meta-analysis of general population cohorts. Lancet..

[8] Tuttle KR, Bakris GL, Bilous RW, Chiang JL, de Boer IH, Goldstein-Fuchs J (2014). Diabetic kidney disease: a report from an ADA consensus conference. Diabetes Care.

[9] Jaisser F, Farman N (2016). Emerging roles of the mineralocorticoid receptor in pathology: toward new paradigms in clinical pharmacology. Pharmacol Rev.

[10] Kolkhof P, Bärfacker L (2017). 30 years of the mineralocorticoid receptor: mineralocorticoid receptor antagonists: 60 years of research and development. J Endocrinol.

[11] Ng K, Arnold J, Sharif A, Gill P, Townend J, Ferro C (2015). Cardiovascular actions of mineralocorticoid receptor antagonists in patients with chronic kidney disease: a systematic review and meta-analysis. J Renin-Angiotensin-Aldosterone System.

[12] Pitt B, White H, Nicolau J, Martinez F, Gheorghiade M, Aschermann M (2005). Eplerenone reduces mortality 30 days after randomization following acute myocardial infarction in patients with left ventricular systolic dysfunction and heart failure. J Am Coll Cardiol.

[13] Pitt B, Zannad F, Remme WJ, Cody R, Castaigne A, Perez A (1999). The effect of spironolactone on morbidity and mortality in patients with severe heart failure. New Eng J Med.

[14] Williams B, MacDonald TM, Morant S, Webb DJ, Sever P, McInnes G (2015). Spironolactone versus placebo, bisoprolol, and doxazosin to determine the optimal treatment for drug-resistant hypertension (PATHWAY-2): a randomised, double-blind, crossover trial. Lancet..

[15] Yancy CW, Jessup M, Bozkurt B, Butler J, Casey DE, Drazner MH, et al. 2013 ACCF/AHA guideline for the management of heart failure. a report of the American College of Cardiology Foundation/American Heart Association task force on practice guidelines. Circulation. 2013;128:e240–327. 10.1161/CIR.0b013e31829e8776 23741058

[16] Yancy CW, Jessup M, Bozkurt B, Butler J, Casey DE, Colvin MM (2017). 2017 ACC/AHA/HFSA focused update of the 2013 ACCF/AHA guideline for the management of heart failure: a report of the American College of Cardiology/American Heart Association task force on clinical practice guidelines and the Heart Failure Society of America. Circulation..

[17] Rossignol P, Dobre D, McMurray JJ, Swedberg K, Krum H, van Veldhuisen DJ (2014). Incidence, determinants, and prognostic significance of hyperkalemia and worsening renal function in patients with heart failure receiving the mineralocorticoid receptor antagonist eplerenone or placebo additional to optimal medical therapy: results from the Eplerenone in mild patients hospitalization and survival study in heart failure (EMPHASIS-HF). Circ Heart Fail.

[18] Sun LJ, Sun YN, Shan JP, Jiang GR (2017). Effects of mineralocorticoid receptor antagonists on the progression of diabetic nephropathy. J Diabetes Invest.

[19] K/DOQI clinical practice guidelines on hypertension and antihypertensive agents in chronic kidney disease (2004). Am J Kid Dis.

[20] KDIGO (2012). Chapter 2: lifestyle and pharmacological treatments for lowering blood pressure in CKD ND patients. Kidney Internat Supplements.

[21] Pitt B, Pedro Ferreira J, Zannad F (2016). Mineralocorticoid receptor antagonists in patients with heart failure: current experience and future perspectives. Eur Heart J Cardiovasc Pharmacother.

[22] Becker GJ, Wheeler DC (2010). Blood pressure control in CKD patients: why do we fail to implement the guidelines?. Am J Kid Dis.

[23] Haller H, Bertram A, Stahl K, Menne J (2016). Finerenone: a new mineralocorticoid receptor antagonist without hyperkalemia: an opportunity in patients with CKD?. Curr Hypertension Rep.

[24] Gosse P, Macfadyen R (2006). Does eplerenone have a future in the management of hypertension in Europe?. J Human Hypertens.

[25] Weinberger MH, Roniker B, Krause SL, Weiss RJ (2002). Eplerenone, a selective aldosterone blocker, in mild-to-moderate hypertension. Am J Hypertension.

[26] Iqbal J, Parviz Y, Pitt B, Newell-Price J, Al-Mohammad A, Zannad F (2014). Selection of a mineralocorticoid receptor antagonist for patients with hypertension or heart failure. Eur J Heart Fail.

[27] K/DOQI. Guideline 11: Use of angiotensin-converting enzyme inhibitors and angiotensin receptor blockers in CKD. Available at: https://kidneyfoundation.cachefly.net/professionals/KDOQI/guidelines_bp/guide_11.htm. Accessed 26 Mar 2019.

[28] Rossignol P, Massy ZA, Azizi M, Bakris G, Ritz E, Covic A, et al. The double challenge of resistant hypertension and chronic kidney disease. Lancet. 2015;386:1588–98. 10.1016/S0140-6736(15)00418-3 26530623

[29] Abbas S, Ihle P, Harder S, Schubert I (2015). Risk of hyperkalemia and combined use of spironolactone and long-term ACE inhibitor angiotensin receptor blocker therapy in heart failure using real-life data: a population-and insurance-based cohort. Pharmacoepidemiology Drug Safety.

