# Supplementary material for: Patient characteristics and initiation of mineralocorticoid receptor antagonists in patients with chronic kidney disease in routine clinical practice in the US: a retrospective cohort study
Source: BMC Nephrol. 2019 May 16;20:171. doi: 10.1186/s12882-019-1348-4 (PMC6524210; doi:10.1186/s12882-019-1348-4)
Supplement: Supplementary file 1 — Table S1. ICD-9-CM pre-inclusion diagnosis codes Table S2. ICD-9-CM codes for comorbidities Table S3. Total healthcare costs (US$) during follow-up by cohort and MRA treatment condition in the CKD Table S4. Treatment and dosage at inclusion date in the MRA cohort. (DOCX 50 kb) [file 12882_2019_1348_MOESM1_ESM.docx]

**Additional file**

**Table S1:** ICD-9-CM pre-inclusion diagnosis codes

| **ICD-9-CM code** | **Chronic kidney disease** |
| --- | --- |
| 585.1 | Chronic kidney disease, stage I |
| 585.2 | Chronic kidney disease, stage II (mild) |
| 585.3 | Chronic kidney disease, stage III (moderate) |
| 585.4 | Chronic kidney disease, stage IV (severe) |
| 585.5 | Chronic kidney disease, stage V |
| 585.6 | End stage renal disease |
| 585.9 | Chronic kidney disease, unspecified |
| 586 | Renal failure unspecified |
| 403.00 | Hypertensive chronic kidney disease, malignant, with chronic kidney disease stage I through stage IV, or unspecified |
| 403.01 | Hypertensive chronic kidney disease, malignant, with chronic kidney disease stage V or end stage renal disease |
| 403.10 | Hypertensive chronic kidney disease, benign, with chronic kidney disease stage I through stage IV, or unspecified |
| 403.11 | Hypertensive chronic kidney disease, benign, with chronic kidney disease stage V or end stage renal disease |
| 403.90 | Hypertensive chronic kidney disease, unspecified, with chronic kidney disease stage I through stage IV, or unspecified |
| 403.91 | Hypertensive chronic kidney disease, unspecified, with chronic kidney disease stage V or end stage renal disease |
| **ICD-9-CM code** | **Heart Failure** |
| 402.01 | Malignant hypertensive heart disease with heart failure |
| 402.11 | Benign hypertensive heart disease with heart failure |
| 402.91 | Unspecified hypertensive heart disease with heart failure |
| 404.00 | Hypertensive heart and chronic kidney disease, malignant, without heart failure and with chronic kidney disease stage i through stage iv, or unspecified |
| 404.01 | Hypertensive heart and chronic kidney disease, malignant, with heart failure and with chronic kidney disease stage i through stage iv, or unspecified |
| 404.02 | Hypertensive heart and chronic kidney disease, malignant, without heart failure and with chronic kidney disease stage v or end stage renal disease |
| 404.03 | Hypertensive heart and chronic kidney disease, malignant, with heart failure and with chronic kidney disease stage v or end stage renal disease |
| 404.10 | Hypertensive heart and chronic kidney disease, benign, without heart failure and with chronic kidney disease stage i through stage iv, or unspecified |
| 404.11 | Hypertensive heart and chronic kidney disease, benign, with heart failure and with chronic kidney disease stage i through stage iv, or unspecified |
| 404.12 | Hypertensive heart and chronic kidney disease, benign, without heart failure and with chronic kidney disease stage v or end stage renal disease |
| 404.13 | Hypertensive heart and chronic kidney disease, benign, with heart failure and chronic kidney disease stage v or end stage renal disease |
| 404.90 | Hypertensive heart and chronic kidney disease, unspecified, without heart failure and with chronic kidney disease stage i through stage iv, or unspecified |
| 404.91 | Hypertensive heart and chronic kidney disease, unspecified, with heart failure and with chronic kidney disease stage i through stage iv, or unspecified |
| 404.92 | Hypertensive heart and chronic kidney disease, unspecified, without heart failure and with chronic kidney disease stage v or end stage renal disease |
| 404.93 | Hypertensive heart and chronic kidney disease, unspecified, with heart failure and chronic kidney disease stage v or end stage renal disease |
| 428.0 | Congestive heart failure unspecified |
| 428.1 | Left heart failure |
| 428.20 | Unspecified systolic heart failure |
| 428.21 | Acute systolic heart failure |
| 428.22 | Chronic systolic heart failure |
| 428.23 | Acute on chronic systolic heart failure |
| 428.30 | Unspecified diastolic heart failure |
| 428.31 | Acute diastolic heart failure |
| 428.32 | Chronic diastolic heart failure |
| 428.33 | Acute on chronic diastolic heart failure |
| 428.40 | Unspecified combined systolic and diastolic heart failure |
| 428.41 | Acute combined systolic and diastolic heart failure |
| 428.42 | Chronic combined systolic and diastolic heart failure |
| 428.43 | Acute on chronic combined systolic and diastolic heart failure |
| 428.9 | Heart failure unspecified |
| **ICD-9-CM code** | **Diabetes Mellitus** |
| 250.00 | Diabetes mellitus without mention of complication, type II or unspecified type, not stated as uncontrolled |
| 250.02 | Diabetes mellitus without mention of complication, type II or unspecified type, uncontrolled |
| 250.10 | Diabetes with ketoacidosis, type II or unspecified type, not stated as uncontrolled |
| 250.12 | Diabetes with ketoacidosis, type II or unspecified type, uncontrolled |
| 250.20 | Diabetes with hyperosmolarity, type II or unspecified type, not stated as uncontrolled |
| 250.22 | Diabetes with hyperosmolarity, type II or unspecified type, uncontrolled |
| 250.30 | Diabetes with other coma, type II or unspecified type, not stated as uncontrolled |
| 250.32 | Diabetes with other coma, type II or unspecified type, uncontrolled |
| 250.40 | Diabetes with renal manifestations, type II or unspecified type, not stated as uncontrolled |
| 250.42 | Diabetes with renal manifestations, type II or unspecified type, uncontrolled |
| 250.50 | Diabetes with ophthalmic manifestations, type II or unspecified type, not stated as uncontrolled |
| 250.52 | Diabetes with ophthalmic manifestations, type II or unspecified type, uncontrolled |
| 250.60 | Diabetes with neurological manifestations, type II or unspecified type, not stated as uncontrolled |
| 250.62 | Diabetes with neurological manifestations, type II or unspecified type, uncontrolled |
| 250.70 | Diabetes with peripheral circulatory disorders, type II or unspecified type, not stated as uncontrolled |
| 250.72 | Diabetes with peripheral circulatory disorders, type II or unspecified type, uncontrolled |
| 250.80 | Diabetes with other specified manifestations, type II or unspecified type, not stated as uncontrolled |
| 250.82 | Diabetes with other specified manifestations, type II or unspecified type, uncontrolled |
| 250.90 | Diabetes with unspecified complication, type II or unspecified type, not stated as uncontrolled |
| 250.92 | Diabetes with unspecified complication, type II or unspecified type, uncontrolled |
| 357.2 | Polyneuropathy in diabetes |

**Table S2:** ICD-9-CM codes for comorbidities

| **ICD-9-CM code** | **Hypertension** |
| --- | --- |
| 401 | Essential hypertension, malignant |
| 401.1 | Essential hypertension, benign |
| 401.9 | Unspecified essential hypertension |
| 402.00 | Malignant hypertensive heart disease without heart failure |
| 402.01 | Malignant hypertensive heart disease with heart failure |
| 402.10 | Benign hypertensive heart disease without heart failure |
| 402.11 | Benign hypertensive heart disease with heart failure |
| 402.90 | Unspecified hypertensive heart disease without heart failure |
| 402.91 | Hypertensive heart disease, unspecified, with heart failure |
| 403.00 | Hypertensive chronic kidney disease, malignant, with chronic kidney disease stage I through stage IV, or unspecified |
| 403.01 | Hypertensive chronic kidney disease, malignant, with chronic kidney disease stage V or end stage renal disease |
| 403.10 | Hypertensive chronic kidney disease, benign, with chronic kidney disease stage I through stage IV, or unspecified |
| 403.11 | Hypertensive chronic kidney disease, benign, with chronic kidney disease stage V or end stage renal disease |
| 403.90 | Hypertensive chronic kidney disease, unspecified, with chronic kidney disease stage I through stage IV, or unspecified |
| 403.91 | Hypertensive chronic kidney disease, unspecified, with chronic kidney disease stage V or end stage renal disease |
| 404.00 | Hypertensive heart and chronic kidney disease, malignant, without heart failure and with chronic kidney disease stage I through stage IV, or unspecified |
| 404.01 | Hypertensive heart and chronic kidney disease, malignant, with heart failure and with chronic kidney disease stage I through stage IV, or unspecified |
| 404.02 | Hypertensive heart and chronic kidney disease, malignant, without heart failure and with chronic kidney disease stage V or end stage renal disease |
| 404.03 | Hypertensive heart and chronic kidney disease, malignant, with heart failure and with chronic kidney disease stage V or end stage renal disease |
| 404.10 | Hypertensive heart and chronic kidney disease, benign, without heart failure and with chronic kidney disease stage I through stage IV, or unspecified |
| 404.11 | Hypertensive heart and chronic kidney disease, benign, with heart failure and with chronic kidney disease stage I through stage IV, or unspecified |
| 404.12 | Hypertensive heart and chronic kidney disease, benign, without heart failure and with chronic kidney disease stage V or end stage renal disease |
| 404.13 | Hypertensive heart and chronic kidney disease, benign, with heart failure and chronic kidney disease stage V or end stage renal disease |
| 404.90 | Hypertensive heart and chronic kidney disease, unspecified, without heart failure and with chronic kidney disease stage I through stage IV, or unspecified |
| 404.91 | Hypertensive heart and chronic kidney disease, unspecified, with heart failure and with chronic kidney disease stage I through stage IV, or unspecified |
| 404.92 | Hypertensive heart and chronic kidney disease, unspecified, without heart failure and with chronic kidney disease stage V or end stage renal disease |
| 404.93 | Hypertensive heart and chronic kidney disease, unspecified, with heart failure and chronic kidney disease stage V or end stage renal disease |
| 405.01 | Secondary renovascular hypertension, malignant |
| 405.09 | Other secondary hypertension, malignant |
| 405.11 | Secondary renovascular hypertension, benign |
| 405.19 | Other secondary hypertension, benign |
| 405.91 | Secondary renovascular hypertension, unspecified |
| 405.99 | Other secondary hypertension, unspecified |
| **ICD-9-CM code** | **Cardiovascular disease** |
| 429.2 | Cardiovascular disease, unspecified |
| 430 | Subarachnoid hemorrhage |
| 431 | Intracerebral hemorrhage |
| 432.0 | Nontraumatic extradural hemorrhage |
| 432.1 | Subdural hemorrhage |
| 432.9 | Unspecified intracranial hemorrhage |
| 433.00 | Occlusion and stenosis of basilar artery without mention of cerebral infarction |
| 433.01 | Occlusion and stenosis of basilar artery with cerebral infarction |
| 433.10 | Occlusion and stenosis of carotid artery without mention of cerebral infarction |
| 433.11 | Occlusion and stenosis of carotid artery with cerebral infarction |
| 433.20 | Occlusion and stenosis of vertebral artery without mention of cerebral infarction |
| 433.21 | Occlusion and stenosis of vertebral artery with cerebral infarction |
| 433.30 | Occlusion and stenosis of multiple and bilateral precerebral arteries without mention of cerebral infarction |
| 433.31 | Occlusion and stenosis of multiple and bilateral precerebral arteries with cerebral infarction |
| 433.80 | Occlusion and stenosis of other specified precerebral artery without mention of cerebral infarction |
| 433.81 | Occlusion and stenosis of other specified precerebral artery with cerebral infarction |
| 433.90 | Occlusion and stenosis of unspecified precerebral artery without mention of cerebral infarction |
| 433.91 | Occlusion and stenosis of unspecified precerebral artery with cerebral infarction |
| 434.00 | Cerebral thrombosis without mention of cerebral infarction |
| 434.01 | Cerebral thrombosis with cerebral infarction |
| 434.10 | Cerebral embolism without mention of cerebral infarction |
| 434.11 | Cerebral embolism with cerebral infarction |
| 434.90 | Unspecified cerebral artery occlusion without mention of cerebral infarction |
| 434.91 | Unspecified cerebral artery occlusion with cerebral infarction |
| 435.0 | Basilar artery syndrome |
| 435.1 | Vertebral artery syndrome |
| 435.2 | Subclavian steal syndrome |
| 435.3 | Vertebrobasilar artery syndrome |
| 435.8 | Other specified transient cerebral ischemias |
| 435.9 | Unspecified transient cerebral ischemia |
| 436 | Acute, but ill-defined, cerebrovascular disease |
| 437.0 | Cerebral atherosclerosis |
| 437.1 | Other generalized ischemic cerebrovascular disease |
| 437.2 | Hypertensive encephalopathy |
| 437.3 | Cerebral aneurysm, nonruptured |
| 437.4 | Cerebral arteritis |
| 437.6 | Nonpyogenic thrombosis of intracranial venous sinus |
| 437.8 | Other ill-defined cerebrovascular disease |
| 437.9 | Unspecified cerebrovascular disease |
| 438.0 | Cognitive deficits due to cerebrovascular disease |
| 438.10 | Unspecified speech and language deficit due to cerebrovascular disease |
| 438.11 | Aphasia due to cerebrovascular disease |
| 438.12 | Dysphasia due to cerebrovascular disease |
| 438.13 | Late effects of cerebrovascular disease, speech and language deficits, dysarthria |
| 438.14 | Late effects of cerebrovascular disease, speech and language deficits, fluency disorder |
| 438.19 | Other speech and language deficits due to cerebrovascular disease |
| 438.20 | Hemiplegia affecting unspecified side due to cerebrovascular disease |
| 438.21 | Hemiplegia affecting dominant side due to cerebrovascular disease |
| 438.22 | Hemiplegia affecting nondominant side due to cerebrovascular disease |
| 438.30 | Monoplegia of upper limb affecting unspecified side due to cerebrovascular disease |
| 438.31 | Monoplegia of upper limb affecting dominant side due to cerebrovascular disease |
| 438.32 | Monoplegia of upper limb affecting nondominant side due to cerebrovascular disease |
| 438.40 | Monoplegia of lower limb affecting unspecified side due to cerebrovascular disease |
| 438.41 | Monoplegia of lower limb affecting dominant side due to cerebrovascular disease |
| 438.42 | Monoplegia of lower limb affecting nondominant side due to cerebrovascular disease |
| 438.50 | Other paralytic syndrome affecting unspecified side due to cerebrovascular disease |
| 438.51 | Other paralytic syndrome affecting dominant side due to cerebrovascular disease |
| 438.52 | Other paralytic syndrome affecting nondominant side due to cerebrovascular disease |
| 438.53 | Other paralytic syndrome, bilateral |
| 438.6 | Alteration of sensations as late effect of cerebrovascular disease |
| 438.7 | Disturbance of vision as late effect of cerebrovascular disease |
| 438.8 | Other late effects of cerebrovascular disease due to cerebrovascular disease |
| 438.81 | Apraxia due to cerebrovascular disease |
| 438.82 | Dysphagia due to cerebrovascular disease |
| 438.83 | Facial weakness as late effect of cerebrovascular disease |
| 438.84 | Ataxia as late effect of cerebrovascular disease |
| 438.85 | Vertigo as late effect of cerebrovascular disease |
| 438.89 | Other late effects of cerebrovascular disease |
| 438.9 | Unspecified late effects of cerebrovascular disease due to cerebrovascular disease |
| **ICD-9-CM code** | **Ischemic heart disease/coronary heart disease** |
| 410.00 | Acute myocardial infarction of anterolateral wall, episode of care unspecified |
| 410.01 | Acute myocardial infarction of anterolateral wall, initial episode of care |
| 410.02 | Acute myocardial infarction of anterolateral wall, subsequent episode of care |
| 410.10 | Acute myocardial infarction of other anterior wall, episode of care unspecified |
| 410.11 | Acute myocardial infarction of other anterior wall, initial episode of care |
| 410.12 | Acute myocardial infarction of other anterior wall, subsequent episode of care |
| 410.20 | Acute myocardial infarction of inferolateral wall, episode of care unspecified |
| 410.21 | Acute myocardial infarction of inferolateral wall, initial episode of care |
| 410.22 | Acute myocardial infarction of inferolateral wall, subsequent episode of care |
| 410.30 | Acute myocardial infarction of inferoposterior wall, episode of care unspecified |
| 410.31 | Acute myocardial infarction of inferoposterior wall, initial episode of care |
| 410.32 | Acute myocardial infarction of inferoposterior wall, subsequent episode of care |
| 410.40 | Acute myocardial infarction of other inferior wall, episode of care unspecified |
| 410.41 | Acute myocardial infarction of other inferior wall, initial episode of care |
| 410.42 | Acute myocardial infarction of other inferior wall, subsequent episode of care |
| 410.50 | Acute myocardial infarction of other lateral wall, episode of care unspecified |
| 410.51 | Acute myocardial infarction of other lateral wall, initial episode of care |
| 410.52 | Acute myocardial infarction of other lateral wall, subsequent episode of care |
| 410.60 | Acute myocardial infarction, true posterior wall infarction, episode of care unspecified |
| 410.61 | Acute myocardial infarction, true posterior wall infarction, initial episode of care |
| 410.62 | Acute myocardial infarction, true posterior wall infarction, subsequent episode of care |
| 410.70 | Acute myocardial infarction, subendocardial infarction, episode of care unspecified |
| 410.71 | Acute myocardial infarction, subendocardial infarction, initial episode of care |
| 410.72 | Acute myocardial infarction, subendocardial infarction, subsequent episode of care |
| 410.80 | Acute myocardial infarction of other specified sites, episode of care unspecified |
| 410.81 | Acute myocardial infarction of other specified sites, initial episode of care |
| 410.82 | Acute myocardial infarction of other specified sites, subsequent episode of care |
| 410.90 | Acute myocardial infarction, unspecified site, episode of care unspecified |
| 410.91 | Acute myocardial infarction, unspecified site, initial episode of care |
| 410.92 | Acute myocardial infarction, unspecified site, subsequent episode of care |
| 411.0 | Postmyocardial infarction syndrome |
| 411.1 | Intermediate coronary syndrome |
| 411.81 | Acute coronary occlusion without myocardial infarction |
| 411.89 | Other acute and subacute form of ischemic heart disease |
| 412 | Old myocardial infarction |
| 413.9 | Other and unspecified angina pectoris |
| [414.00](http://www.icd9data.com/2015/Volume1/390-459/410-414/414/414.00.htm) | Coronary atherosclerosis of unspecified type of vessel, native or graft |
| 414.01 | Coronary atherosclerosis of native coronary artery |
| 414.02 | Coronary atherosclerosis of autologous vein bypass graft |
| 414.03 | Coronary atherosclerosis of non-autologous biological bypass graft |
| 414.04 | Coronary atherosclerosis of artery bypass graft |
| 414.05 | Coronary atherosclerosis of unspecified bypass graft |
| 414.06 | Coronary atherosclerosis of native coronary artery of transplanted heart |
| 414.07 | Coronary atherosclerosis of bypass graft (artery) (vein) of transplanted heart |
| 414.10 | Aneurysm of heart (wall) |
| 414.11 | Aneurysm of coronary vessels |
| 414.12 | Dissection of coronary artery |
| 414.19 | Other aneurysm of heart |
| 414.2 | Chronic total occlusion of coronary artery |
| 414.3 | Coronary atherosclerosis due to lipid rich plaque |
| 414.4 | Coronary atherosclerosis due to calcified coronary lesion |
| 414.8 | Other specified forms of chronic ischemic heart disease |
| 414.9 | Chronic ischemic heart disease, unspecified |
| **ICD-9-CM code** | **Left ventricular hypertrophy** |
| 429.3 | Cardiomegaly |
| **ICD-9-CM code** | **Anemia** |
| 280.9 | Iron deficiency anemia, unspecified |
| 281.1 | Other vitamin B12 deficiency anemia |
| 281.2 | Folate-deficiency anemia |
| 281.3 | Other specified megaloblastic anemias not elsewhere classified |
| 281.4 | Protein-deficiency anemia |
| 281.9 | Unspecified deficiency anemia |
| 283.10 | Non-autoimmune hemolytic anemia, unspecified |
| 283.11 | Hemolytic-uremic syndrome |
| 283.9 | Acquired hemolytic anemia, unspecified |
| 284.01 | Constitutional red blood cell aplasia |
| 284.09 | Other constitutional aplastic anemia |
| 284.11 | Antineoplastic chemotherapy induced pancytopenia |
| 284.12 | Other drug-induced pancytopenia |
| 284.19 | Other pancytopenia |
| 284.2 | Myelophthisis |
| 284.81 | Red cell aplasia (acquired)(adult)(with thymoma) |
| 284.89 | Other specified aplastic anemias |
| 284.9 | Aplastic anemia, unspecified |
| 285.0 | Sideroblastic anemia |
| 285.1 | Acute posthemorrhagic anemia |
| 285.21 | Anemia in chronic kidney disease |
| 285.22 | Anemia in neoplastic disease |
| 285.29 | Anemia of other chronic disease |
| 285.3 | Antineoplastic chemotherapy induced anemia |
| 285.8 | Other specified anemias |
| 285.9 | Anemia, unspecified |
| **ICD-9-CM code** | **Edema** |
| 518.4 | Acute edema of lung, unspecified |
| 581.9 | Nephrotic syndrome with unspecified pathological lesion in kidney |
| 782.3 | Edema |
| **ICD-9-CM code** | **Proteinuria** |
| 791.0 | Proteinuria |
| **ICD-9-CM code** | **Hypoglycemia** |
| 251.0 | Hypoglycemic coma |
| 251.1 | Other specified hypoglycemia |
| 251.2 | Hypoglycemia, unspecified |
| 270.3 | Disturbances of branched-chain amino-acid metabolism |
| **ICD-9-CM code** | **Hyperkalemia** |
| 276.7 | Hyperpotassemia |

**Table S3:** Total healthcare costs (US$) during follow-up by cohort and MRA treatment condition in the CKD population

| **Treatment condition** | **Variable** | **CKD** | **DKD** | **CKD+HF** | **DKD+HF** |
| --- | --- | --- | --- | --- | --- |
| **No MRA treatment** | *n* | 112,730 | 75,616 | 14,653 | 21,144 |
|  | Mean | 24,768 | 33,649 | 63,905 | 81,880 |
|  | 95% CI | 24,408 to 25,127 | 33,149 to 34,149 | 61,995 to 65,815 | 80,148 to 83,612 |
|  | SD | 61,578 | 70,181 | 117,973 | 128,481 |
|  | Median | 7,473 | 13,144 | 26,359 | 38,885 |
| **Spironolactone < 6 months** | *n* | 869 | 891 | 512 | 808 |
|  | Mean | 50,806 | 55,497 | 107,673 | 108,834 |
|  | 95% CI | 43,964 to 57,648 | 49,546 to 61,447 | 93,587 to 121,759 | 98,312 to 119,356 |
|  | SD | 102,762 | 90,506 | 162,241 | 152,373 |
|  | Median | 18,015 | 26,176 | 51,479 | 66,910 |
| **Spironolactone ≥ 6 months** | *n* | 481 | 469 | 373 | 458 |
|  | Mean | 33,242 | 39,917 | 89,953 | 80,321 |
|  | 95% CI | 28,067 to 38,418 | 33,726 to 46,108 | 76,527 to 103,379 | 71,781 to 88,861 |
|  | SD | 57,765 | 68,227 | 131,863 | 93,002 |
|  | Median | 11,960 | 18,020 | 49,198 | 51,525 |

*CI* confidence interval, *CKD* chronic kidney disease, *DKD* diabetic kidney disease, *HF* heart failure, *MRA* mineralocorticoid receptor antagonist, *SD* standard deviation

**Table S4:** Treatment and dosage at inclusion date in the MRA cohort

|  | **CKD** | | **DKD** | | **CKD+HF** | | **DKD+HF** | |
| --- | --- | --- | --- | --- | --- | --- | --- | --- |
|  | (*n* = 1,574) | | (*n* = 1,574) | | (*n* = 1,004) | | (*n* = 1,747) | |
| **Treatment, *n* (%)** | | | | | | | | |
| Spironolactone | 1,511 | (96.0) | 1.520 | (96.6) | 973 | (96.9) | 1,702 | (97.4) |
| Eplerenone | 63 | (4.0) | 54 | (3.4) | 31 | (3.1) | 45 | (2.6) |
| **Spironolactone dose, mg^a^** | | | | | | | | |
| Mean | 40.7 | | 37.1 | | 32.4 | | 33.4 | |
| 95% CI | 38.9 to 42.6 | | 35.6 to 38.6 | | 30.5 to 34.4 | | 32.2 to 34.7 | |
| SD | 35.3 | | 28.5 | | 29.8 | | 26.2 | |
| Median | 25.0 | | 25.0 | | 25.0 | | 25.0 | |
| Range | 0.03 to 40 | | 5 to 300 | | 5 to 450 | | 1.79 to 400 | |
| **Eplerenone dose, mg^a^** | | | | | | | | |
| Mean | 41.7 | | 43.6 | | 34.3 | | 31.7 | |
| 95% CI | 36.3 to 47.1 | | 37.4 to 49.9 | | 21.4 to 47.1 | | 26.6 to 36.8 | |
| SD | 21.4 | | 22.6 | | 35.1 | | 16.4 | |
| Median | 50.0 | | 50.0 | | 25.0 | | 25.0 | |
| Range | 12.5 to 100 | | 12.5 to 100 | | 12.5 to 200 | | 12.5 to 100 | |

^a^ Based on average daily dose at inclusion, calculated as strength times quantity divided by days’ supply

*CI* confidence interval, *CKD* chronic kidney disease, *DKD* diabetic kidney disease, *HF* heart failure, *SD* standard deviation
